# Friedrich Edelhäuser: Wahrnehmen und Bewegen – Grundlagen einer allgemeinen Bewegungslehre

**DOI:** 10.3205/zma001584

**Published:** 2023-02-15

**Authors:** Christian Scheffer

**Affiliations:** 1 Universität Witten/Herdecke, Integriertes Begleitstudium Anthroposophische Medizin, Witten, Germany

## Bibliographical details

Friedrich Edelhäuser

**Wahrnehmen und Bewegen – Grundlagen einer allgemeinen Bewegungslehre**

Kohlhammer Verlag, Stuttgart

Year of publication: 2022, 198 pages, prizes: € 39,00

ISBN: 978-3-17-036270-3

## Review

How does a human being move? Are his actions an expression of his primary motor cerebral cortex or do they reflect the intentions of a human individuality? How do we see? Are we passive recipients or do we have to actively shape our seeing? What results from approaching these questions by bringing together scientific and phenomenological-philosophical insights? Can guiding viewpoints for person-centered medicine be gained from this? 

These questions are illuminated comprehensively and in many ways in the newly published book *“Wahrnehmen und Bewegen – Grundlagen einer allgemeinen Bewegungslehre”* (Perceiving and Moving - Foundations of a General Theory of Movement) by Friedrich Edelhäuser. In doing so, the author takes us to astonishing phenomena, to reflections worth considering, and to profound questions. 

Using the example of looking at a mountain landscape, the first phenomena of seeing are looked at: our gaze goes inwardly through the picture, searching for various objects and contours and arranging the details into a meaningful overall context. What at first appears to be fixed thus becomes experienceable as a process. 

In the “objectifying” view of physiology, vision is characterized as a process akin to a camera in which light passes through a lens onto the retina and then leads to electrochemical nervous processes. In this process, the qualitative perceptions melt down into a measurable but qualityless process. Vision thus becomes an example of the stimulus-response sequence, in which an external sensory stimulus becomes electrochemical processes inside, i.e. in the brain, and is answered with a reaction. Thereby not only the quality of the perceived disappears, but also the perceiving person. In the following, this so-called third-person-perspective as an objectifying approach is supplemented by introspection, the first-person-perspective. 

In chapter 5 “perceiving and moving” the process of seeing is examined more closely. In doing so, one becomes aware of the fact that seeing includes an inner scanning of the contour to be perceived. This unconscious movement of the eyeballs can be represented technically and shows individual movement patterns, similar to gait or handwriting. If this movement is suppressed, the perceived blurs to a gray-in-gray for a short time due to the lack of contrast. During further analysis, it is noticeable that one is not only aligned to the object to be seen with one's eye muscles, but with one's entire head and body posture. Only this self-movement makes seeing possible. Something similar can be shown for hearing and other sensory modalities. 

Looking back at the mountain landscape, it becomes clear that there is a circular relationship, the perception of the image and the contours that can be found in it are guiding the scanning movements of the eye, which in turn are conditions for what is to be seen. Thus, there is no monocausal relationship with temporal succession, but a mutually dependent one. 

In chapter 6 the Gestalt circle of W. V. Weizsäcker is introduced and the mutual enabling of perceiving and moving is examined in more detail. The organism-environment relationship is constituted in an encompassing, circular process of perception and movement. Intentional attention is now examined as a further object of investigation. On the basis of optical examples, in which one can see different things in the same form context depending on intention (e.g. either a transparent cube oriented to the front or to the back), it becomes clear that perception is not something simply given or depicted, but something given up, something produced by a directed, intentional act of perception. This intentional attention influences my seeing in different ways, so it can change between center and periphery, between foreground and background, between future and past. The sensually given is not unambiguous in the way it appears, but is accessible to ambiguous interpretations. 

Chapter 7 now turns to human movement. In the sense of the stimulus-response model, one assumes here in the traditional conceptions a purely efferent process, in which from the center, the brain, a movement impulse goes over motor nerves to the target musculature and triggers the movement. Here, too, the book comes up with exciting phenomena. For example, the case of Ian Waterman, who, at the age of 19, falls ill with a viral infection and within a few days loses both his sense of touch and proprioception, and thus his sense of position and orientation of his limbs, from the neck down. Thus, he also loses the ability to move at the same time, since this requires not only an efferent nervous structure, but also a perception of the context of movement. The patient solves this with an extraordinary achievement by compensating the missing sense of movement by the sense of sight in a laborious learning process and by controlling the movement by seeing the limb movements. From this – and from specific experiments – it becomes clear that a movement that is meaningfully placed in context only becomes possible through a complex interplay of efferent and afferent processes, which at the same time require the intentional shaping of the subject. Thus, moving presupposes perceiving and vice versa. The intentional shaping processes are called self-movement, following Aristotle. 

Both, perceiving and moving, are thus circularly causally connected processes, which do not function meaningfully without the respective other part. At the same time, it becomes clear that individual intentionality becomes effective in the way in which perceiving is controlled via self-movement and moving is controlled via perception. Thus, not only the brain or the nervous system is an expression of individuality, but also the organization of movement. This basic idea is continued in the further chapters with Rudolf Steiner’s functional threefold structure as well as with modern embodiment research, which is concerned with the question of how the entire body is in resonance with the experience and intentions of an individuality. 

Specific therapeutic-physiological issues are also addressed, including the question of how everyday movements as well as meditative forms of movement such as therapeutic eurythmy affect cardiovascular regulation. 

The book spans a wide range from everyday phenomena to exciting case presentations and reflection on one's own perceptual processes to very fundamental philosophical and anthropological questions. This is at times demanding, but at the same time fruitful and stimulating. 

Is there a need for such a book in the education of physicians and further health professionals? I would have wished for such a book in my medical education, in which physiological processes are presented so clearly and brought into the context of fundamental anthropological questions. The otherwise frequent separation into “objective” physiology, which leaves out the inner human being, and a philosophical approach, which at the same time leaves out the basic science conditions, are here brought together in an exciting, easily comprehensible and illuminating way. From this can be derived not only helpful points of view for a medicine that perceives the individuality of a human being in the intentionality of his perceptions and movements, in his self-movement and in his entire bodily expression, but also for teaching. For here, too, we often have a passive image of the learners, who primarily have to absorb content, while we give insufficient space to the inner intentional self-movement of the students. 

## Competing interests

The author declares that he has no competing interests.

